# Was the Watchmaker Blind? Or Was She One-Eyed?

**DOI:** 10.3390/biology6040047

**Published:** 2017-12-20

**Authors:** Raymond Noble, Denis Noble

**Affiliations:** 1 Institute for Women’s Health, University College London, Gower Street, London WC1E 6BT, UK; r.noble@ucl.ac.uk; 2 Department of Physiology, Anatomy & Genetics University of Oxford, S Parks Rd, Oxford OX1 3QX, UK

**Keywords:** blind chance, harnessing stochasticity, hypermutation, evolutionary hold mechanisms, adaptability driver, internal and external goals

## Abstract

The question whether evolution is blind is usually presented as a choice between no goals at all (‘the blind watchmaker’) and long-term goals which would be external to the organism, for example in the form of special creation or intelligent design. The arguments either way do not address the question whether there are short-term goals within rather than external to organisms. Organisms and their interacting populations have evolved mechanisms by which they can harness blind stochasticity and so generate rapid functional responses to environmental challenges. They can achieve this by re-organising their genomes and/or their regulatory networks. Epigenetic as well as DNA changes are involved. Evolution may have no foresight, but it is at least partially directed by organisms themselves and by the populations of which they form part. Similar arguments support partial direction in the evolution of behavior.

## 1. Introduction

We again use our computer monkey, but with a crucial difference in its program. It again begins by choosing a random sequence of 28 letters, just as before ... it duplicates it repeatedly, but with a certain chance of random error—‘mutation’—in the copying. The computer examines the mutant nonsense phrases, the ‘progeny’ of the original phrase, and chooses the one which, *however slightly,* most resembles the target phrase, METHINKS IT IS LIKE A WEASEL. (Richard Dawkins, *The Blind Watchmaker*).

### 1.1. Background

In chapter 3 of his book, *The Blind Watchmaker* [1], Richard Dawkins produces his famous Weasel program. He shows that a monkey writing out 28 characters randomly on a typewriter would require much more than the whole lifetime of the universe to arrive by pure chance at a correct 28 letter sequence to match Shakespeare’s text [2]. But if each correct character were to be held constant between successive generations of random typing, it would require only a modest number (43) of iterations to achieve a correct result. The program resembles the operation of an old-fashioned three-wheel fruit (slot) machine. If the target for a reward is, say, three lemons and a spin of the wheels produces two, the best strategy might be to hold the wheels with the two lemons and spin the remaining wheel until that also shows a lemon. The number of ‘wheels’ (28 in the Weasel example) doesn’t change the principle of this mechanism.

The example shows that, however unlikely a pattern might be, it might evolve in a reasonable amount of time by using such an incremental strategy. 

Dawkins acknowledges that the original program does not truly represent the process of blind variation followed by natural selection assumed in neo-Darwinist evolutionary models since it uses a long-term goal set by the computer program writer. The program only ‘knows’ when to hold a character constant between generations by comparing it with the long-term goal. As Dawkins writes:

“Life isn’t like that. Evolution has no long-term goal. There is no long-distance target, no final perfection to serve as a criterion for selection, although human vanity cherishes the absurd notion that our species is the final goal of evolution. In real life, the criterion for selection is always short-term, either simple survival or, more generally, reproductive success.”

It is also important to acknowledge that more complex versions of the Weasel program have been produced that do not require that a correct character should be completely fixed. In those cases back-mutation is also possible. But all these programs still require various kinds of comparison, in the selection process, with the long-term goal in order to succeed. When we refer to the ‘hold’ metaphor in this article it is important to note that this does not completely exclude mutations. It refers to the ability to preserve existing functionality sufficiently well for subsequent generations to inherit.

A further important deficiency in the Weasel program as originally formulated is that it assumes that the goal would be correctly represented by a particular genome sequence. From the viewpoint of organisms and their functionality, that is not correct. Genomes and phenotypes are far from being equivalent. The mismatch works both ways. The same genome can be used to generate many different phenotypes and the same phenotype can evolve through many different genome variations, to such an extent that the sequence variations may even be unpredictable [3]. It is of course the functional phenotype that is ‘seen’ by natural selection. DNA sequences are not directly available for selection other than through their functional consequences in the production of RNAs and proteins, and even most of those variations are effectively buffered by the regulatory networks and so may also be invisible to natural selection. For example, 80% of DNA knockouts in yeast are ‘silent’ in controlled experimental conditions [4]. We will return to this important point later (see Section 4.2) since it is the fundamental reason why gene-centric views of evolution are incorrect. Evolution is a high-level *forming* process, not simply a matter of genome informatics.

### 1.2. Purpose and Organization of This Article

In this article we will agree with Dawkins that (a) completely stochastic processes with no ‘hold’ or similar ‘guiding’ mechanism would require impossibly long periods of time for successful evolution to occur, and (b) there is no need to assume that evolution has a long-term goal. This is where both he and we part company with Intelligent Design (ID) and creationist theories. 

But we will nevertheless show that organisms and populations of organisms do have identifiable and empirically testable goals, and that variations on the theme of the Weasel program found experimentally in nature, show this to be true. The key to understanding why we differ from neo-Darwinists on this matter lies in multi-level feedback processes that have been shown to exist which enable organisms and populations to direct their evolution in response to stimuli from the environment and so achieve the inheritance of acquired characteristics. These feedback processes require analysis of function at a high (systems) level, e.g., networks, cells, tissues, organs and the organism as a whole in interaction with the environment, including other organisms. Multi-level feedback is a requirement of goal-directed behaviour. A purely gene-centric view will not necessarily ‘see’ such feedback. Empirical tests used routinely in physiology and engineering do so readily. 

Our article is not intended to be a systematic review. It is rather the development of a conceptual interpretation of the process of evolution that differs from neo-Darwinism in implementing the principle that there is no privileged level of causation [5,6]. We believe this is a novel conceptual advance. It differs radically from views of evolution that privilege the role of DNA sequences in the intergenerational transmission of inheritance [7,8,9], and is therefore more sympathetic to views of evolution that emphasise the active role of functional regulatory networks and behavior in organisms [6,10]. These are the processes that endow organisms with what we will call natural purposiveness. DNA alone cannot do that. Outside the regulatory network environment of the complete cell it is inactive. 

We develop our case in stages: first, to show how multicellular organisms use targeted evolution of their cells to respond to environmental challenge; second, to show how populations of microorganisms achieve similar targeted responses; third, to show how epigenetic inheritance occurs in multicellular organisms with separate germ-lines; fourth; to show how the evolution of behavior can use similar processes that have developed agency in their evolution. In all these cases, variation is not random with respect to genome location and/or organism functionality. The targeting of variation and the preservation of existing functionality ensure that evolution is not entirely blind. Where relevant we reference alternative viewpoints, including standard neo-Darwinist interpretations. But this article does not analyse where we believe those viewpoints are deficient. That was the purpose of a recent related article [11].

## 2. Definitions

Agency: an agent acts, it does not just react in the way, for example, in which a billiard ball is caused by another ball to move. Organisms are agents to the extent that they can interact socially with other organisms to choose particular forms of behavior in response to environmental challenges. This definition of agency can therefore apply to microorganisms, such as bacterial films and eukaryotic slime moulds, that form interacting communities [12,13] as well as to multicellular organisms. In principle, it can also apply to the subcellular networks responsible for buffering organisms against many forms of DNA variation.

Goals: Goals can be ascribed to agents since choice of action involves directionality in their actions. A goal in this sense is the situation towards which the agent’s action leads. Goals arise naturally from within the agent’s cognitive behavior, albeit in interaction with other agents. This kind of behavior can be called natural purposiveness. Goals can therefore be ascribed empirically on the basis of observation of the behavior of organisms.

Teleology: The possession of goals is what defines teleology. Some biologists prefer the word teleonomy [14] to emphasise the view that goals in organisms (sometimes with the qualification ‘other than humans’) are only apparent. Since our use of the word ‘goal’ enables empirical physiological tests for the presence of the required natural purposiveness we see no need to avoid the word teleology. 

Natural purposiveness: Natural purposiveness is an emergent property of multi-level evolved systems. It is easier to understand and appreciate its significance within the principle of biological relativity, i.e., no privileged level of causation [6].

Neo-Darwinism: Classical neo-Darwinism was formulated by August Weismann [15,16] and others in the late nineteenth century to expunge the inheritance of acquired characteristics from Darwin’s theory. Blind variation followed by natural selection was claimed to be *entirely sufficient* (Weismann’s *allmacht*). This is clear from his extensive argument with Herbert Spencer [17,18,19]. Many biologists today redefine neo-Darwinism in various ways (see e.g., the on-line dialogue between one of us and David Sloan Wilson (https://thebestschools.org/dialogues/evolution-denis-noble-david-sloan-wilson/) and the relevant entry in the Encyclopedia of Evolution [20]). Redefining a term does not however change the fact that the original theory using that term is no longer the complete story. Our position can therefore, to some degree, be seen to return to Darwin’s multi-mechanism viewpoint, though with vastly extended empirical evidence (see Figure 6 in reference [11]). 

Gene-centrism: We will refer to gene-centric views of evolution several times in this article. There are two senses in which we view neo-Darwinist theories as gene-centric. The first is the view that the genome is “the Book of Life” [21], i.e., that the development of an organism is essentially a read out of the DNA sequences, in interaction with the environment. The hidden assumption here is that inheritance depends on DNA alone. Sometimes this is spelt out, as in the distinction between the ‘replicator’ (DNA) and the ‘vehicle’ (the rest of the ‘disposable’ organism). The second sense is that, even though it is the phenotype that is selected in evolution, only those aspects of the phenotype that are represented in DNA are inherited. 

These definitions are conceptual, as are all definitions, but they endow the theory we develop here with empirically testable predictions. 

## 3. Goals within Organisms

### 3.1. Regulated (Directed) Hypermutation Processes

The Weasel program example shows that the monkey at the keyboard needs some kind of guidance to have any chance at all of reaching the goal. In the evolutionary process the ‘monkey at the keyboard’ is blind chance mutations, the process assumed in neo-Darwinism to be the only process producing genetic variation. The assumption that all mutations are produced by blind chance is central to the theory. This is the assumption that appears to exclude goal-directed behavior [6]. 

Yet, as we will show in this paper, organisms have demonstrably evolved *guided* random mutation mechanisms that can respond rapidly and correctly to environmental challenges. These mechanisms allow organisms and populations to harness stochasticity to evolve a solution to such challenges at high speed compared to what could be achieved by blind chance alone. It is the harnessing of stochasticity in guided response to environmental challenges that achieves what blind chance alone could not possibly do [11]. 

One way in which the guidance can occur is through the process of natural selection. Progressively, through the generations, selection acts as a filter. Neo-Darwinism assumes that this is the only guide. We disagree with that view because it is demonstrably insufficient: nature also uses other faster guidance processes.

How can that be achieved? The answer is already implicit in our fruit machine analogy. The quickest way to achieve the fruit machine target is to hold correct wheels while spinning the others to let chance find the target. By analogy, this is precisely what the immune system does within our bodies. 

**Figure 1:**
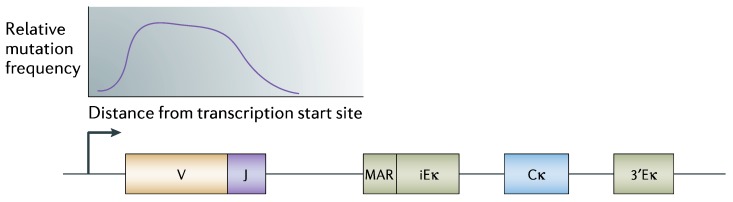
Schematic diagram of gene-specific targeted hyper-mutation in immunoglobulin gene loci. The mutation rate is greatly increased only in the variable part of the genome, which is a ~1.5 kilobase region in each of the three immunoglobulin loci. In this figure, the rectangular elements (V, J, MAR, iEκ, Cκ, 3′Eκ) represent different functional parts of the DNA sequence for the immunoglobulin protein. V is the variable part, subject to hypermutation, while the other parts are fixed. For further details on the functions of the parts see Odegard and Schatz [22]. Those details are not important for the purposes of this article.

Figure 1 summarizes how this is achieved. Faced with a new antigen challenge, the mutation rate in the variable part of the genome can be accelerated by as much as 1 million times. So far as is known, those mutations all occur stochastically. But the location in the genome is certainly not a matter of chance. The functionality in this case lies precisely in the specific targeting at the relevant part of the genome. The mechanism is directed, because the arrival of the antigen itself activates the hypermutation process, and its binding to a successful antibody triggers proliferation of those cells that make it. What this mechanism achieves is that all the other ‘wheels’ in the DNA sequence forming a template for the immunoglobulin protein are held sufficiently constant for functionality to be retained. Even more remarkably, all the functionality in the rest of the genome is also maintained. Considering the huge size of the complete genome, this is pin-point targeting requiring highly specific feedback processes to be successful.

### 3.2. Is the System Purposive?

Holding correct parts of the immunoglobulin sequence constant is the way rapid mutation can then be restricted to only very small *and relevant* parts of the whole genome. Hyper-mutation of all the immunoglobulin sequence, and even more so everywhere in the genome, would not work. As Odegard and Schatz say:

“Somatic hypermutation (SHM) introduces mutations in the variable region of immunoglobulin genes at a rate of ~10^−3^ mutations per base pair per cell division, which is 10^6^-fold higher than the spontaneous mutation rate in somatic cells. To ensure genomic integrity, SHM needs to be targeted specifically to immunoglobulin genes.”

What this example shows is that the basic idea in Dawkins’ Weasel program is actually broadly correct. Imagine that the monkey already has XYZHINKS IT IS LIKE A WEASEL. Then the best strategy is to treat only the XYZ sequence with stochastic mutation until MET turns up. Within the Weasel program analogy, it would be essential to hold the sequence HINKS IT IS LIKE A WEASEL constant. 

At this point it is important to recall what we emphasized in the INTRODUCTION: Evolution is a high-level forming process, not simply a matter of genome informatics. The more correct way to look at the process therefore is that it is the high-level functionality that corresponds to HINKS IT IS LIKE A WEASEL and to any equivalently functional sequence that needs to be maintained. Any low-level sequence changes that are neutral with respect to phenotype functionality would not matter. The targeting may therefore be attributable to higher-level buffering by regulatory networks in addition to differential genome mutation rates. This point is important since not all the examples we discuss later in this article necessarily involve differential rates of mutation.

This is also why it is misleading to talk of the ‘language of the genes’ [23] or the ‘book of life’ [21]. In a language, the sequence *is* the written language’s ‘phenotype’. That is even more obvious in languages employing idiograms. By contrast, the genome is a template resource used by the organism, and is far from identical with or simply translatable into the phenotype.

The targeted mechanism in the immune system has been known and intensely studied for many years [24]. So, how did many people not realise that it is a physiologically guided process? The answer is that the guidance does not lie at the genome level. At the genome level the process appears blind. It depends on stochastic mutation. The functionality enabling the process to be described as guided lies in the system as a whole. 

The system includes: (a) sensing the environmental challenge, i.e., the antigen invasion, (b) transmitting this signal to the nuclei of immune system cells to trigger hyper-mutation in just a tiny fraction of the genome. (c) Then sensing of the correctness or otherwise of the outcome, followed by the “reproduce or die” signal: cells that do not produce an antibody that fits the antigen do not survive. At this stage, natural selection occurs amongst the population of immune system cells [25]. This is a complete finely-tuned physiological feedback system that rapidly generates an acquired characteristic in response to an environmental challenge, which is then inherited in the surviving population of cells. This is what is *meant* by a goal-oriented system. By all the usual criteria this is a teleological, i.e., goal-directed, process (see Section 2). 

It may not be perfect; it doesn’t have to be. Not all keys have to be perfect to open a lock. The system feels its way forward, harnessing stochasticity to create novelty while using targeted preservation of what already works. The targeted preservation is what gives the system its purpose: to maintain its own integrity. It uses stochasticity to change what it must change, precisely because that is the part that doesn’t work. 

It is important moreover to see that the goal, the directionality, exists *within* the organisms and their populations. The goals of organisms have developed during the evolutionary process. Our position does not therefore require the ideas of Intelligent Design. In agreement with this aspect of Dawkins’ position, we do not have to assume there is a long-term goal. 

At this stage it is also important to clarify that we partly agree with alternative (such as neo-Darwinian) views of hypermutation mechanisms, to the extent of saying that such differential mutation rates must have evolved, and that the neo-Darwinian mechanism of stochastic variation combined with natural selection has operated [26,27,28,29,30,31]. The point to understand is that, once hypermutation has evolved and is linked to environmental feedback that endows the organism with natural purposiveness, subsequent evolution is not purely neo-Darwinian. Natural purposiveness evolves and then changes the nature of subsequent evolution. There is a transition, one of many transitions in evolution [32], the most spectacular of which has been the transition to enable cultural evolution leading to the development of humans, to which we will return in Section 6. 

### 3.3. Natural Genetic Engineering

Such physiologically functional feedback leading to genomic change in response to an environmental challenge is not restricted to the immune system. In fact, responsiveness of the genome generally to environmental stress was discovered by the Nobel laureate, Barbara McClintock, more than 70 years ago. Working on Indian corn, she showed that in response to stress genetic material can move around even between different chromosomes [33]. She was therefore the discoverer of what are now called mobile genetic elements, known more colloquially as ‘jumping genes’. In her 1983 Nobel Prize lecture she wrote:

“In the future attention undoubtedly will be centered on the genome, and with greater appreciation of its significance as a *highly sensitive organ of the cell*, monitoring genomic activities and correcting common errors, sensing the unusual and unexpected events, and *responding to them, often by restructuring the genome*. We know about the components of genomes that could be made available for such restructuring. We know nothing, however, about how the cell senses danger and instigates responses to it that often are truly remarkable” (our italics).

[34]

This was highly perceptive since it was written before whole genome sequencing. By 2001 with the publication of the first complete draft of the human genome, it became possible to compare genome sequences in different organisms. The results show that movements of whole domains of sequences corresponding to functional domains of transcription factor proteins and chromatin proteins must have occurred as evolution diverged to produce organisms as different as worms, yeast, flies, mouse and human [6,24,35].

Movement and rearrangement of functional domains of proteins can also function as a mechanism for speeding up evolutionary change. Like targeted hypermutation it also avoids having to wait for very slow accumulation of small (point) mutations. To appreciate this in less technical language, imagine two children given Lego bricks to construct a model bridge. To the first child we give a pile of the original small Lego bricks which have to be laboriously pieced together to form an architectural feature like an arch. To the second child we give some preformed Lego structures. It is obvious that the second child will construct a realistic bridge much faster than the first. 

Moving complete functional domains around the genome is therefore a bit like the mirror image of hypermutation since it recombines already functional parts of proteins. In terms of the Weasel program, imagine already having METHINKS IT IS and LIKE A WEASEL. Joining them up is worth trying. Of course, not all joined up sequences will produce new functionality. What the mechanism gives is a much improved chance of obtaining new functionality. There is a bias in the process, which is precisely the extent to which it is not blind. It plays with existing functionality. As we will show later in this paper, behavioural evolution can use comparable mechanisms in which existing functionality is preserved and rearranged.

## 4. Goals within Populations

The cells of the immune system can evolve extremely rapidly to achieve the goal of the system, where the goal is the protection of the organism, and the system is the organism itself. But each organism does not transmit all of this information to its progeny. In this section we will look at ways in which populations of organisms can use stochastic mutation to evolve inheritable functional responses to environmental challenges very rapidly. 

### 4.1. Contingency Loci in Bacteria

A comparable mechanism to that employed by the immune system has been extensively investigated by Richard Moxon and his colleagues who use the term ‘contingency locus’ to characterise the targeted loci of hypermutable DNA [36]. In bacteria, these loci are simple sequence repeats in which the repeating unit is one to several nucleotides in length. In eukaryotes these loci are called microsatellites and often consist of hundreds of repeats. In both kinds of organism these microsatellites are prone to high rates of mutation through slippage during strand repairing, leading to either increases or decreases in the number of repeat units. When these mutations occur within functional gene sequences, they can therefore produce high frequency reversible switching of genotype. Since “mutation rates vary significantly at different locations within the genome” they propose that “it is precisely in the details of these differences and how they are distributed that major contributions to fitness are determined.” In an earlier article, Moxon and Thaler write

“This phenotypic variation, which is stochastic with respect to the timing of switching but has a programmed genomic location, allows a large repertoire of phenotypic solutions to be explored, while minimizing deleterious effects on fitness.”.

[37]

Moxon and Thaler’s conclusion is correct. If the hyper-mutation were not restricted to a small subset of the genes, the results would certainly be deleterious, just as non-targeted hypermutation of immunoglobulin genes would rapidly destroy the functional proteins of the immune system. 

The phenotype effects can also be combinatorial. The example given by Moxon et al. [36] is that switching in just seven independent loci to produce two genotypes in each case could generate up to 128 phenotypes. Many of these phenotype changes occur significantly in bacterial cell surface structure, which is the structure through which organisms detect changes in the environment and foreign invaders. They can also generate switching between metabolic and regulatory cell networks: Ritz et al. discovered a triplet repeat enabling *E. coli* to switch between adaptation to reducing and oxidizing environments [38].

Can such processes be demonstrated in actual evolutionary time? An example of organisms making use of this ability to reorganise their genomes is the study of Bos et al., who have observed the emergence of antibiotic resistance from multinucleated bacterial filaments. They write:

“The strategy of generating multiple mutant chromosomes within a single cell may represent a widespread and conserved mechanism for the rapid evolution of genome change in response to unfavorable environments (i.e., chemo-therapy drugs and antibiotics)”.

[39]

Similarly, Jack et al. (2015) have shown that

“Signaling pathways that sense environmental nutrients control genome change at the ribosomal DNA. This demonstrates that not all genome changes occur at random and that *cells possess specific mechanisms to optimize their genome in response to the environment*.” (our italics).

[40]

It is important at this stage in the argument to note that, in addition to the functional feedback, an essential property in purposive genome adaptation in response to environmental stress is the ‘hold’ mechanism, by which existing functionality is preserved. This mechanism can operate whether or not hypermutation comparable to that in the immune system and many bacteria occurs. Hypermutation is simply an extreme example of the non-random location of mutations. Any differential mutation rate in genomes *might* be exploited by organisms and so improve their chances of generating new functionality, though equally clearly differential mutation rates alone do not *necessarily* indicate functionality. The relevant feedback loops with environmental interaction must also exist. That is an essential part of how a goal-oriented system is defined (see Section 2). The evolution of such links is a major transition.

### 4.2. Genetic Buffering by Regulatory Networks

**Figure 2:**
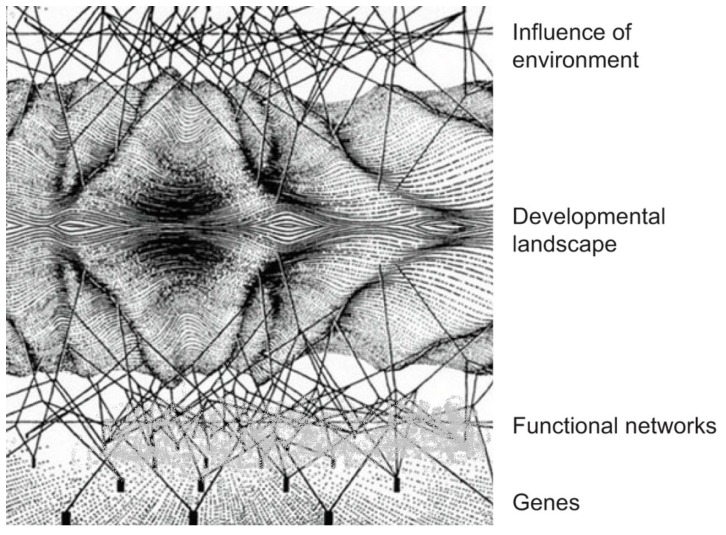
Development of Waddington’s (1957) landscape diagram [43]. The original diagram was simply the lower half of this diagram, which Waddington used to indicate that the developmental phenotype (the landscape) is not directly dependent on the genes (the pegs at the bottom) but also depends on the regulatory networks represented as lying in between genes and the phenotype. Our version of the diagram incorporates two new features. First, organisms are open systems sensitive to the environment. This is represented by the top half of the diagram. Second, the regulatory networks in the lower half (the original half) of the diagram act to buffer genetic variation. This is represented by a ‘cloud’ covering a large fraction of the genome, corresponding to the fact that many mutations at the genome level are silent functionally. The regulatory networks can buffer many variations at the genome level. The filtering action of the ‘cloud’ performs a function similar to that of the ‘hold’ mechanism in this article. Differential mutation rates are not therefore essential to enable organisms to guide their own evolution.

Buffering of genome variation by regulatory networks may also be involved. This is a further important part of our argument so we have represented it in a development of Waddington’s famous landscape diagram shown in Figure 2. As in the original diagram, genes (as DNA sequences) are represented by the pegs at the bottom. The regulatory networks are represented as lying between the genes and the phenotype. Genes can only influence the phenotype through the networks; they do not do so directly. They themselves do not exhibit agency in the sense in which we define it. This is one of the reasons why we believe the ‘selfish gene’ metaphor is misleading. Moreover, from a physiological perspective, selfish gene theory is not testable [41].

We have added two new features to the diagram. The first is the inclusion of environmental interactions above the phenotype landscape. The second is a cloud which we have placed to represent buffering of genomic change by the networks. Inspired by Hillenmeyer’s work on yeast [4], we have represented the cloud as covering as much as 80% of the genome, which is the proportion of silent knockouts in Hillenmeyer’s experiments, to which we have already referred in the INTRODUCTION. Similar robustness has been found in the networks involved in the natural pacemaker of the heart, where multiple mechanisms exist that can maintain rhythm if one is disabled either by genetic change or by pharmacological blockers [42].

Of course, that percentage will vary with different species, cell types and many other parameters. The buffering cloud may cover different proportions of genomic change under different environmental and experimental conditions. Thus, Hillenmeyer et al.’s experiments to which we referred earlier [4] also showed 97% of genes to be functional by varying metabolic conditions. The controlled experimental conditions in which 80% were silent are unlikely to match actual wild population conditions. The cloud is therefore dynamically variable, as its name suggests. Note also that the cloud is not a separate structure from the networks. It represents the filtering (buffering) action of the networks: a process of the networks, not an object in its own right.

The idea of the cloud mechanism was already implicit in Waddington’s early work on what he called epigenetics [43] and has been confirmed by many physiological and developmental studies since then on the robustness of organisms in accommodating genomic variations, the most recent studies being the comparative failure of genome-wide association studies to reveal very much about the genetic origins of health and disease [44,45]. This is one of the most important empirical findings arising from genome sequencing. But its implications for evolutionary biology have not yet been sufficiently well appreciated. 

The main implication that is relevant to this article is that the hold mechanism need not require targeted differential mutation rates. In the current stage of our knowledge we do not believe it is possible to estimate how often evolutionary change might depend on targeted differential mutation compared to the operation of regulatory networks protecting themselves via ‘cloud’ mechanisms. Those mechanisms are of course a further aspect of the robustness of regulatory networks in the face of genetic changes. Nor are the mechanisms only epigenetic. The phenomenon of epistasis by which different genes can influence each other’s effects can play a similar role, particularly when the interactions, mediated through the networks, are to cancel each other’s effects [46].

### 4.3. Switch of Function in Regulatory Networks

Networks can not only act as buffers, they can also switch function. Our next example is from the work of Taylor et al. [47] who have shown that bacteria that have lost their flagella through deletion of the relevant DNA sequence can evolve the regulatory networks required to restore flagella and so restore motility in response to a stressful environment within just four days.

That ability is a property of the bacterium regulatory networks and of the ability of the organism to signal the environment pressure to those physiological networks to enable them to adapt. It is that feedback that makes such a rapid and clearly functional response possible. Two mutations were involved:

“Step one mutations increase intracellular levels of phosphorylated NtrC, a distant homolog of FleQ, which begins to commandeer control of the FleQ regulon at the cost of disrupting nitrogen uptake and assimilation. Step two is a switch-of-function mutation that redirects NtrC away from nitrogen uptake and toward its novel function as a flagellar regulator. Our results demonstrate that natural selection can rapidly rewire regulatory networks in very few, repeatable mutational steps”.

Viewed from the level of the genome site(s) where the mutations are occurring, there is a process of Darwinian selection amongst the results of stochastic mutation, as the authors themselves say. But the response clearly exploits the physiological regulatory properties of *existing* cell regulatory networks, resulting in a switch of function at the regulatory network level. 

### 4.4. Roles of Stochasticity and Natural Selection

This is also a suitable point at which to emphasise that we are not denying the essential contribution of a neo-Darwinian process, i.e., mutation followed by natural selection. Natural selection necessarily operates within the context of these evolved functional characteristics. The key to our argument lies in the way in which organisms maintain and develop existing functionality *either* through exploiting differential mutation rates *or* through buffering the effects of mutations in many parts of the genome, *or* combinations of the two processes. It requires a multi-level systems-level approach to see that the neo-Darwinist process is harnessed, so that it is not sufficient in itself to explain the functionality of what is happening. 

This point reflects once again our insistence that evolution is a high-level forming process. It may help to clarify that there are two senses in which this point has force. The first is conceptual. Even when a process is entirely neo-Darwinian when viewed from the genome sequence level, that viewpoint is not necessarily the most productive way to view it. Much more than the genome is inherited. The roles of regulatory networks, which are relatively well buffered against sequence changes, and the physiological feedback processes that ensure that the process leads to the inheritance of a characteristic in response to an environmental change, are better characterized from a physiological systems perspective. They also function within a cellular structural environment involving lipids and other components not coded for by the genome. 

The second sense is empirical. It may, as a matter of fact, be the case that at the molecular level targeted differential mutation rates form an essential part of the process that explains the speed with which the adaptation occurs. In this article we have given examples of both of these senses. 

### 4.5. Communication to the Genome

How can genomes know about what is happening at the cell surface? The physiological mechanisms by which events in tiny micro-domains near the cell surface signal to the nucleus, and so control specific gene expression levels, have now been studied in fine detail [48,49]. There is no longer any mystery in understanding the highly specific transmission of information to the nucleus that can control gene expression. There is no reason why genomes should not use similar communication pathways in response to stress signals received by cells and organisms. 

## 5. Speculation 1: Goals Achieved through Epigenetic Inheritance

### 5.1. Different Forms of Epigenetics

Epigenetics was originally introduced and defined by Conrad Waddington [43] to refer to the role of networks in organisms in interpreting and controlling their genetic inheritance. Waddington was a developmental biologist and he correctly identified the general mechanisms by which development, in interaction with the environment and genes, could canalize both development itself, and subsequently also inheritance, towards specific phenotypes. His experiments on fruit flies showed that selection for environmentally-induced variants could become assimilated into the genome within a relatively small number of generations, as few as around 14 or 15. In so doing, he produced one of the first examples of the inheritance of acquired characteristics based on rigorous multigenerational experiments.

In addition to Waddington’s mechanism of genetic assimilation of acquired characteristics, there was the discovery of transcription factors, i.e., proteins that convey signals to the genome from higher level networks to control levels of gene expression. This mechanism is what makes it possible for cells as different as bone cells and heart cells to be developed using the same genome. In vertebrates, around 200 clearly distinct cell types are produced in this way during development and in the maintenance of tissue types in the adult. 

Recently, the mechanisms of epigenetic control of the genome have been greatly extended, through the discoveries of DNA marking, histone marking, and many processes by which the germline can be either side-stepped [50] or itself marked, or modified by the transmission in the germline of functional RNAs [51,52]. 

### 5.2. Experimental Examples

These developments are transforming the study of genetics in evolution. As just one well-documented example, Michael Skinner and his colleagues have experimented on one of the icons of Darwinian evolution, the Galapagos finches, to find that there are as many epigenetic as genetic variations and that the number of epigenetic variations between the species correlate rather better with phylogenetic distance between them than do the genetic variations [53]. But it will be almost impossible to determine which came first in the evolution of the different species, since epigenetic and genetic changes necessarily interact. Moreover, some authors have highlighted the important role that ‘soft’ (epigenetic) inheritance can play in evolutionary change [54,55].

Given that organisms are active agents and can choose their behavior in response to the environment (including other organisms), they must be able to mark their genomes with variations as a consequence of their behavior. This is the mechanism described by Michael Meaney and his colleagues in showing how stroking behavior in rodents can mark the genome in the hippocampus to predispose the young to adopt the same affective behavior as adults [56].

Similarly, many life-time choices can now be shown to act epigenetically to influence health and disease in subsequent generations. Hanson and Skinner have written a valuable review of these effects [57]. They include all the evidence for environmentally-induced inheritable epigenetic impacts shown in Table 1, taken from their review. 

Regional differences and the dramatic increases in disease frequencies are hard to explain without recourse to epigenetic mechanisms, just as identical twin studies show that genome differences alone are insufficient.

All of the epigenetic effects referred to in this section are well-documented experimentally (see Menger [58] and Skoblov et al. [59] for further examples). We come now to our first main speculation. If organisms have agency and, within obvious limits, can choose their lifestyles, and if these lifestyles result in inheritable epigenetic changes, then it follows that organisms can at least partially make choices that can have long-term evolutionary impact. 

### 5.3. Role in Speciation

We refer to this as a speculation because, as Skinner says, it may be very difficult to disentangle epigenetic and genetic changes using estimations based on the differences between living species. Ideally, we would need to document both genetic and epigenetic changes as a function of time during the developments that led to the speciation. Given the long periods of time over which speciation occurs, it is difficult to see how such experiments could ever be performed. Like astronomers, we usually have to infer the past from what we observe now. Moreover, as Waddington showed, epigenetic changes can become assimilated into the genome: what begins as ‘soft-wired’ may become ‘hard-wired’. One of the mechanisms by which such assimilation may occur is analysed in Noble, reference [6] (pp. 216–219) which is also the mechanism Waddington himself proposed. More generally epigenetic changes produce changes in function and behaviour that lead to organisms choosing different niches. Genetic change can then follow. That process itself would usually ‘hide’ evidence of what initiated the change.

Exceptionally, the speciation process can be sufficiently rapid to be observed during very few generations. This is so for the remarkable case of an immigrant finch to one of the islands in the Galápagos archipelago which initiated a new genetic lineage by breeding with a resident finch. Genome sequencing of the immigrant identified it as a male that belonged to another species more than 100 km from the island. To quote the paper:

“From the second generation onwards the lineage bred endogamously, and despite intense inbreeding, was ecologically successful and showed transgressive segregation of bill morphology. This example shows that reproductive isolation, which typically develops over hundreds of generations, can be established in only three.”.

[60]

The rapidity with which this reproductively separate line was established is expected since one of the most important triggers for animal and plant speciation is interspecific hybridization. An important feature of hybrid germlines is disruption of normal epigenetic control which translates into increased genome restructuring and mobile element activity. Thus, in addition to forming novel combinations of genome components, hybridization triggers genome innovation by modification of a higher-level control regime, as outlined in more detail in the review by Shapiro [61].

## 6. Speculation 2: The Evolution of Goal-Directed Behaviour

The effect of behavioural control on evolutionary change could be especially great when the social environment is a major component of the challenges faced by animals. The result would be that individuals would evolve to understand and predict what other members of the social group are about to do [62].

### 6.1. The Continuity of Animal and Human Evolution

This article is itself part of the proof of what we are leading up to. We and the imagined monkeys at their keyboards in the Weasel example evolved from common hominoid ancestors several million years ago. They in turn evolved from the ancestors of all mammals, in turn from the pre-Cambrian fauna, and so on as we stretch back to the last eukaryotic ancestor, or even the last universal common ancestor, maybe 3 billion years ago. Unless we are to return to a theory of special creation, humans capable of writing this article form a complete evolutionary continuum with the whole of the rest of life on earth. 

It is implausible to suppose that goal-directedness and creative purpose suddenly appeared only with the first humans [63]. It is therefore important to identify the roots of such mechanisms that have evolved in other organisms. If goal-directedness is identified as the ability to anticipate what other organisms may do and then act on that ability, then there is no serious difficulty in observing that many organisms in addition to humans can do this [10,62,64,65,66,67,68]. Animals like monkeys, dogs and wolves are sensitive to inequity in the behaviour of others [66,69] and so can favour the formation of cooperative groups. An important criterion is the ability to show unlimited associative learning, which is necessary for such anticipatory and innovative behavior. Bronfman et al. show that this can be observed in animals ranging from vertebrates to arthropods and cephalopods [70]. 

We contend that neo-Darwinism doesn’t have the conceptual tools necessary to even begin to understand the transition to goal-directedness and creative purpose since it assumes a priori that these do not exist, they are only ‘apparent’. By insisting on the necessarily exclusive role of blind chance in generating novelty it ignores the fact that such chance has to be, and has been, harnessed by organisms, which are therefore also *active* agents in their own development and evolution [62,71]. As Bateson says so succinctly:

“the picture of the external hand of natural selection doing all the work is so compelling that it is easy to regard organisms as if they were entirely passive in the evolutionary process.”.

(p. 105)

If we really insist on the passive role of the organism, we will not even recognize the significance of active agency in evolution. Assuming that natural selection is the only directing process is equivalent to denying that organisms have agency, or at least that, if they do, it does not play any role in their evolution. Darwin would not have agreed. He clearly identified the role of sexual selection in evolution [72]. 

### 6.2. The Adaptability Driver

Novelty, creativity, can only emerge if stochasticity is harnessed, rather than given free rein. Stochasticity everywhere is destructive, not constructive. But this requires the progressive building-in of mechanisms that include various forms of ‘hold’ when partial solutions have already been found. This is how organisms come to ‘know’ [73] what to keep and what to reject. Of course, all of this ‘knowledge’ is built on the processes of stochasticity and selection. Equally clearly, those processes on their own are insufficient. Furthermore, what becomes built-in includes much more than the genome since it also includes regulatory networks and 3D membranous systems that are also inherited and without which DNA is inactive. This is yet another example of our point that evolution is a high-level forming process, not simply a matter of genome informatics.

Can we therefore generalize the targeted mutation examples to apply not only to physical but also to behavioural evolution and the various forms of active role that organisms may play in their own evolution? 

That is surprisingly easy. Targeted mutation allows stochasticity to be harnessed precisely because the organism’s use of it is not blind. On the contrary, it is linked to higher-level processes that enable the organism to be an agent (see Section 2). The organism combines mutation with buffering of all that it is important to retain. Change is always combined with preservation. That ability is active in the sense that the knowledge required to identify what to preserve and what to change originates within the organism itself. How it originally evolved to have that knowledge is important, but not immediately relevant to the case being made here. Like the emergence of attractors in physical systems, once they have emerged, the clock can’t be turned back. 

Very early in the development of Darwin’s theory of evolution it was noticed that organisms have the adaptability to choose or even create new niches for themselves and so to partially direct their own evolution. Darwin himself drew attention to the idea as it applied to sexual selection [72], where it is clearly true that choices of mate according to desired characteristics would influence future evolution. Once again, we quote Bateson on this aspect:

“Charles Darwin (1871) argued that choice of a mate could drive evolution. He called the evolutionary process ‘sexual selection’. Alfred Russel Wallace, although the co-author with Darwin of the first clear statement about the role of Natural Selection, did not like the new idea. Indeed, for many years most biologists did not take sexual selection seriously. When I was an undergraduate I was told confidently that, even if it were possible in theory, the process probably played little part in biological evolution. In recent years, however, many experiments have supported Darwin’s thinking.”.

[67]

The generalization of the idea of organism choice in evolution was originally attributed to Baldwin and became known as the Baldwin effect [74,75]. Bateson has researched the history and development of the idea [76]. The process was first identified by Douglas Spalding in 1873 [77]. To avoid historical confusion and to let the name of the process be easier to understand it, Bateson chose to call it the *adaptability driver*. We like this nomenclature for two reasons. First, adaptability is the behavioural equivalent of the targeted mutation process. The key lies in the restriction of change only to *parts* of the behavioural repertoire. Second, the word ‘driver’ captures the active role of the organism itself. Blind chance is then not the only driver of novelty. 

### 6.3. The Role of Contextual Logic in the Behavior of Organisms

We will conclude this article with a brief sketch of how contextual logic enters into the behaviour of organisms so that they do not react purely passively, but rather become active agents in interaction with their environment. From an evolutionary perspective, organisms have become active agents behaviourally because there are obvious advantages in being able to anticipate the behavior of other organisms. For a predator, anticipating the behavior of prey may be the difference between lunch and no lunch. Conversely, for prey, to be able to anticipate the behaviour of a predator could be the difference between death and survival. Such anticipation assumes rule-guided behavior by organisms for prediction and anticipation to be possible. What results is like a game. Iterative game-playing is clearly not unique to humans. 

We may then extend the analogy with the processes of targeted genome mutation and re-organisation outlined earlier in this article. Winning games, including those between predator and prey, depends precisely on a combination of preservation and change. On the one hand, organisms learn the repetitive rules of interaction. The better those rules are known, the more effective will be the organism’s anticipation of the behavior of others. On the other hand, innovation requires the ability to break out from the rules, to foil the anticipation of others. We speculate that this is where stochasticity plays a role similar to that of the harnessing of stochasticity in targeted genome variation. Organisms that can combine rule-guided anticipation with occasional innovative behavior will have a selective advantage. Our speculation is that selective harnessing of stochasticity enables innovation, just as it enables targeted genome variation, but the benefits depend also on combining innovation with conservation of routine behavior. 

Once organisms have acquired such ability, they become active agents, and all the well-known evolutionary consequences of the ‘adaptability driver’ then follow. 

## 7. Conclusions

### 7.1. The Harnessing of Stochasticity

In this article we have outlined mechanisms by which organisms and populations harness stochasticity and so improve their chances of developing functional responses to environmental challenges. Provided that we correctly interpret the targeted nature of genome variation and its necessary correlate, i.e., the preservation of already functional genome sequences and/or the buffering of genome change by the regulatory networks, the principle of Dawkins’ Weasel program becomes broadly correct. The ‘hold’ mechanism corresponds to the spatial targeting of mutation or the buffering of many genetic variations by the active networks: all other sequences and network properties are sufficiently well preserved (‘held’) to maintain functionality. 

Some evolutionary biologists have attributed what we describe as an active ‘hold’ mechanism to a passive ‘ratchet’ mechanism [32,78,79,80,81]. A process now known as Muller’s Ratchet was introduced in 1964 following a series of papers in which he explored the evolutionary significance of sex. He showed mathematically that the damage that would result from accumulation of deleterious mutations can be ameliorated by recombination, either by exchange of DNA between organisms or by recombination through sexual reproduction. Muller’s Ratchet idea captures a form of blind directionality (ratchets go forwards not backwards) in evolution but it does not capture the agency of organisms themselves which is implied by the ‘hold’ metaphor. 

Maynard Smith and Szathmary included the ratchet mechanism in their 1995 book on *The Major Transitions in Evolution* [32]. A further advance in this idea was described by Lukeš et al. [79] to demonstrate how a ratchet process which, following Stoltzfus [82] they term Constructive Neutral Evolution can generate cellular complexity. They come close to our idea of the functional significance of genetic buffering (represented as the cloud in Figure 2) when they write “The interaction (between two biochemical components, A and B), though not under selection, permits (suppresses) mutations in A that would otherwise inactivate it.” Our reading of “permits (suppresses)” is precisely the cloud mechanism. While their paper is an undoubted advance on the ratchet idea, it does not include a targeting of the process of mutation, nor does it include feedback from and to the environment. 

This is the reason why functionally significant ‘hold’ mechanisms forming parts of purposive feedback systems should not be interpreted simply as a ‘ratchet’ mechanism. It is precisely the targeted nature of the complete physiological feedback system *using* the ‘hold’ mechanism that generates functionality and gives the subsequent evolutionary process a direction, driven by organisms themselves. To repeat what we wrote earlier in Section 3, “this is what is *meant* by a goal-oriented system.” 

### 7.2. Organisms as Agents

Yet, our interpretation of the correctness of the Weasel program uses a mechanism that Dawkins did not himself acknowledge. The reason is that neo-Darwinists generally eschew the concepts of goal-directedness or teleology. The idea of active agency in organisms runs counter to neo-Darwinist thought. We suggest that this view is motivated partly by what are perceived as the dangers of misinterpretation of teleological explanations, which are thought to support the ideas of creationism and intelligent design. But attributing agency and directedness to organisms themselves makes no commitment whatsoever to ideas of long-term goals in evolution. We therefore believe that this concern is misplaced. In any case, the scientific investigation of goal-directed behavior should not be restricted by perceived opportunities for misinterpretation. 

This also explains why classical neo-Darwinism [15,16], and modern versions of it [1,8] completely excluded the inheritance of acquired characteristics (see Section 2). Such inheritance is sometimes thought to open the door to theories of long-term external directionality in evolution. Confusingly also, this kind of directionality is often associated with Lamarck and his concept of “le pouvoir de la vie” [83]. This phrase was mistakenly interpreted to become identified with ‘vital force’, which is a serious misreading of Lamarck’s writing [84,85]. Lamarck was a thorough-going materialist and was opposed to the vitalists of his time. As the French historian of genetics André Pichot writes:

‘Lamarck’s claim that … there is a radical difference between living beings and inanimate objects might lead people to think that he was a vitalist. But he is not. On the contrary, his biology is a mechanistic reply to the physiological vitalism of Bichat, which was then the dominant theory’.

[86]. (Our translation of Pichot’s French)

Lamarck’s “pouvoir de la vie” would therefore be better interpreted as an innate tendency in organisms to evolve in a directed way [87]. This is precisely what the mechanisms described in this article achieve. The directionality is innate in organisms and populations as active agents.

We have also shown that some forms, at least, of behavioural evolution can be interpreted within the same scheme of harnessed stochasticity combined with preservation of existing functional forms. 

In an important article analyzing many aspects of this issue in the context of a review of Dawkins’ *The Extended Phenotype*, Eva Jablonka covers some of the points we make here, including this quotation:

Being scared of Lamarckism leads to the neglect of the evolutionary effects of evolved systems that allow the inheritance of targeted and acquired variations. When the fact that variation is highly constrained and is shaped (or, rather, drafted) by the rules of the generating system is ignored, evolution cannot be properly understood.

[88]

To which we can add that this neglect is inhibiting the development of a fully integrated physiological (i.e., functional) interpretation of evolutionary biology [6]. Some of the problems with neo-Darwinist interpretations arise from conceptual limitations, including privileging causation from the molecular genetic level, for which there is no empirical justification [89]. One of the aims of our article is to encourage empirical investigations of the ideas we put forth. Theories of goal-directedness can be tested, and engineers and physiologists have the tools to do so. 

### 7.3. Organisms and Their Populations Are the One-Eyed Watchmakers

We return now to where this article began: *The Blind Watchmaker* and the monkey at the keyboard. The Watchmaker analogy is a powerful one, both for those who follow Paley’s original use of it to argue for creationism or intelligent design, and for Dawkins’ use of it to argue for nothing but blind chance. But consider this: the only watchmakers we know are organisms (humans). They evolved from other organisms. The ability to be a watchmaker therefore evolved. There is therefore nothing surprising in the fact that goal-directed agency occurs also in other organisms and is capable of influencing evolution.

Our overall conclusion is that there are several processes by which directed evolutionary change occurs—targeted mutation, gene transposition, epigenetics, cultural change, niche construction and adaptation. Evolution is an ongoing set of iterative interactions between organisms and the environment. Evolution is a continuous organic process. Directionality is introduced by the agency of organisms themselves as the one-eyed watchmakers. Evolution itself also evolves [90].

## Figures and Tables

**Table 1:** Environmental epigenetic impacts on biology and disease.

• Worldwide differences in regional disease frequencies
• Low frequency of genetic component of disease as determined with genome wide association studies (GWAS)
• Dramatic increases in disease frequencies over past decades
• Identical twins with variable and discordant disease frequency
• Environmental exposures associated with disease
• Regional differences and rapid induction events in evolution
